# Comparison of methods for faecal hormone preservation and analysis in African savanna elephants under field conditions

**DOI:** 10.1093/conphys/coaf026

**Published:** 2025-04-23

**Authors:** Daniella E Chusyd, Emily Chester, Tessa Steiniche, Stephanie Dickinson, Bailey Ortyl, Steve Paris, Nicole Boisseau, Michael Wasserman, Janine L Brown

**Affiliations:** Department of Environmental and Occupational Health, Indiana University, 2719 E. 10th St., Bloomington, IN 47408, USA; College of Veterinarian Medicine, Auburn University, 1130 Wire Rd., Auburn, AL 36849, USA; Department of Environmental and Occupational Health, Indiana University, 2719 E. 10th St., Bloomington, IN 47408, USA; Department of Epidemiology and Biostatistics, Indiana University, 1025 E. 7th St., Bloomington, IN 47405, USA; Department of Epidemiology and Biostatistics, Indiana University, 1025 E. 7th St., Bloomington, IN 47405, USA; Center for Species Survival, Smithsonian National Zoo & Conservation Biology Institute, 1500 Remount Rd., Front Royal, VA 22630, USA; Center for Species Survival, Smithsonian National Zoo & Conservation Biology Institute, 1500 Remount Rd., Front Royal, VA 22630, USA; Department of Anthropology, Indiana University, 701 E. Kirkwood Ave., Bloomington, IN 47405, USA; Center for Species Survival, Smithsonian National Zoo & Conservation Biology Institute, 1500 Remount Rd., Front Royal, VA 22630, USA

**Keywords:** Faecal hormones, field research, glucocorticoids*, Loxodonta africana*progesterone, thyroid

## Abstract

Noninvasive faecal hormone analyses can provide valuable information on the physiological state of wild animals and how they respond to ecological changes or anthropogenic disturbances. However, preservation techniques to prevent hormone alteration can be problematic, and not all are field friendly. We compared five processing methodologies to preserve samples for faecal glucocorticoid, progestagen and thyroid hormone metabolites. Samples were collected from adult zoo Africa savanna elephants (*Loxodonta africana)* (one male, four females) immediately after defecation. Subsamples were then subjected to five preservation methods: lyophilisation (LYO) (considered the gold standard), dehydration, solid-phase extraction (SPE) and two ethanol extraction methods—with and without being immediately dried down. Faecal glucocorticoid, progestagen and thyroid hormone metabolites were quantified by validated enzyme immunoassays. After 7 days at room temperature (to emulate shipping conditions), faecal glucocorticoid metabolite concentrations were lower for all methods compared to LYO. For thyroid hormone metabolite concentrations, the dehydration process resulted in higher concentrations compared to LYO, whereas with SPE, concentrations were lower. For faecal progestagen metabolite concentrations, there were no discernible differences across methods. Based on these results, we recommend ethanol extraction followed by immediate sample desiccation, a method that combines technical simplicity with the advantage of ambient temperature sample storage and transportation. Nevertheless, each investigator should consider the best method for the research question, field conditions, budget, equipment accessibility and shipping requirements, especially as results can vary by species and assay used. With growing interest in assessing animal welfare, validating field methods for noninvasive hormone monitoring is essential.

## Introduction

Noninvasive hormone analysis is a valuable conservation tool for monitoring welfare and reproduction in free-ranging animal populations (Viljoen *et al.,* 2008; Freeman *et al.,* 2010). Faecal sampling is particularly useful because it is noninvasive and, therefore, causes the least interference and disturbance to the individuals being monitored. A number of studies have used faecal monitoring to assess stress and reproductive hormones in African savanna elephants (*Loxodonta africana*) in relation to land use and responses to human disturbance (Wasser *et al.,* 1996; Foley *et al.,* 2001; Gobush *et al.,* 2008; Oduor *et al.,* 2020; Pokharel and Brown, 2023). With elephant population numbers declining in many regions and interactions with humans increasing (Chase *et al.,* 2016), the continued use of noninvasive hormone monitoring is invaluable to understanding how changing environments are associated with elephant physiology, welfare and, ultimately, survival. However, field conditions can make it challenging to preserve, store and ship elephant faecal samples to laboratories for hormone determinations, either in-country or overseas.

After defecation, alteration due to microorganism metabolism can change the analytes in samples. To stop the breakdown of steroid metabolites, samples need to be frozen within a few hours (Hunt and Wasser, 2003; Palme, 2005; Crossey *et al.,* 2018; Webster *et al.,* 2018). Field-friendly methods for progestagen and, recently, glucocorticoid metabolites have been developed for elephants (Freeman *et al.,* 2010; Lacomme *et al.,* 2023), although resources and logistical considerations were varied. While some field sites have access to freezers and are outfitted with standard laboratory equipment (e.g. centrifuge, pipettes, enzyme immunoassay [EIA] equipment), this is not always the case, particularly for remote camps. Further, most preservation studies have focused on only one hormone of interest, but the field is increasingly pushing for more comprehensive studies, necessitating the analyses of multiple hormones from one sample. Thus, the goal of this study was to test different field-friendly methods for stabilizing African savanna elephant faecal samples for measuring glucocorticoid (fGCM), progestagen (fPM) and thyroid (fT3) metabolites to provide options and flexibility to investigators working under different field site conditions.

## Materials and Methods

### Study animals and sample collection

**Figure 1:**
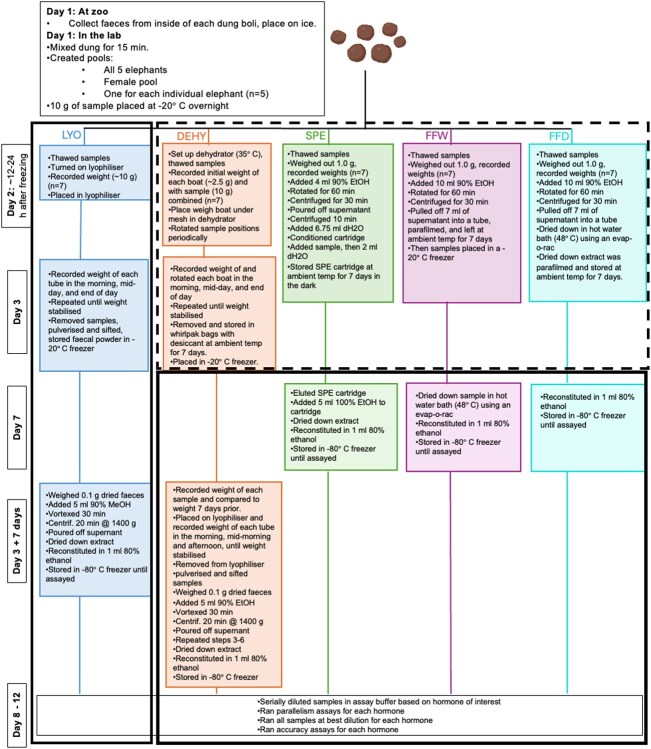
Methodological details for each preservation method with its respective timeline. Except for LYO, samples sat for 7 days at ambient temperature after each respective preservation method before storage at a minimum of −20°C. Dotted lined box indicates work that would theoretically occur in the field, while solid lined boxes indicate work that would theoretically occur in the laboratory.

Single faecal samples were collected from one male (16 years of age) and four female (15, 40, 46 and 46 years of age) African savanna elephants residing at the Indianapolis Zoo. Immediately after an observed defecation, elephants were moved out of the area to permit sample collection. Approximately 2 kg of faecal material were collected from each elephant, collecting samples from inside each faecal bolus. Samples were placed into a labelled bag and kept in a cooler on ice until transported to the laboratory within 4 hours of defecation, after which samples were mixed by hand for a minimum of 15 minutes, and 10 g were weighed and subsampled into 50-ml tubes for each of the five preservation techniques (Fig. 1). All samples were stored for 12–24 hours at −20°C until processing.

### Preservation technique

The processing steps for each subsample for each preservation technique are detailed (Fig. 1), separated by which steps would theoretically be done in the field versus in the laboratory. As lyophilisation (LYO) serves as the gold standard, and having a lyophilizer in the field is typically not feasible, all aspects of LYO were treated as if they would occur in the laboratory.

### Lyophilisation

Adapted from Hunt and Wasser (2003), in the laboratory, wet samples (*n* = 7) were lyophilized (Labconco, Kansas City, MO) until the weight stabilized (24–26 hours). Dried samples were pulverized using a mortar and pestle and sifted to obtain faecal powder, which was then stored at −20°C for 7 days. Then, approximately 0.1 g (±0.01 g) of dried faecal powder was weighed and placed into a 16-mm borosilicate tube with 5 ml of 90% methanol. Samples were shaken on a multitube vortexer (VWR multitube vortexer, 58 816–115, Troemner, USA) for 30 minutes at speed 6 in a fume hood and then centrifuged (Eppendorf, Centrifuge 5702) for 20 minutes at 2400*g*. The supernatant was poured into a clean tube, and an additional 5 ml of 90% methanol was added to the pellet, which was then vortexed for 60 seconds and centrifuged for 20 minutes at 715 g. The supernatants were combined, dried down at 50°C using an Evap-o-Rac (Cole Parmer, Vernon Hills, IL) and then reconstituted in 1 ml of 80% ethanol. Faecal extracts were stored at −80°C until hormone analyses, which occurred within 5 days.

### Dehydration

#### Procedures to be conducted in the field

Adapted from Lacomme *et al.* (2023), wet faecal samples were thawed, and approximately 10 g (±0.1 g) were spread out on aluminium weigh boats covered with aluminium mesh screens and placed into a dehydrator (Nesco FD-75PR) set to 35°C. Samples were rotated 90° three times at periodical intervals throughout the drying process. Samples were reweighed at approximately 2, 20, 25 and 27 hours until weights stabilized. Dried samples were placed into a whirl-pak bag (S-19794, Uline, Pleasant Prairie, WI) with desiccant packs and stored in a dark place at ambient temperature for 7 days to replicate shipment conditions.

#### Procedures to be conducted in the laboratory

After 7 days, the dry faecal samples were reweighed and lyophilized again as weights increased slightly (~0.03 g/sample). Weights stabilized after 3 hours in the lyophilizer. Dried faecal samples were pulverized using a mortar and pestle and then sifted to collect the faecal powder, and approximately 0.1 g (±0.01 g) of dried faecal powder was used for extractions. Faecal extractions were similar to LYO samples, except 90% ethanol rather than 90% methanol was used. Ethanol was used as it is easier to obtain and dispose of in range countries than methanol in the event this step is done in the range country. Faecal extracts were dried, reconstituted in 1 ml of 80% ethanol and then stored at −80°C until hormone analyses within 5 days.

### Solid-phase extraction

#### Procedures to be conducted in the field

Adapted from Edwards *et al.* (2014), wet faecal samples were thawed, and approximately 1 g (±0.01 g) of wet faeces were weighed and placed into a 15-ml falcon tube. Four millilitres of 90% ethanol was added to each tube and rotated for 60 minutes at 30 rpm and then centrifuged for 30 minutes at 2400*g*. The supernatants were poured into clean tubes and centrifuged again for 10 minutes, followed by adding 6.75 ml of ultra-pure water to each sample. Cartridges (Thermo Scientific Hypersep C8 SPE column 500 mg/3 ml, product # 60108-309) were primed by pushing through 4 ml of deionized water at 1 ml/minute, followed by 4 ml of 100% ethanol at 1 ml/minute using a vacuum. The sample was then loaded onto the cartridge at a rate of 1 ml/2 minutes, and 2 ml of deionized water was pushed through at a rate of 1 ml/minute. The cartridge was sealed with parafilm and stored in a dark place at ambient temperature for 7 days.

#### Procedures to be conducted in the laboratory

The solid-phase extraction (SPE) cartridge was eluted using a vacuum manifold, slowly passing through 5 ml of 100% ethanol. The extract was dried using an Evap-o-rac and then reconstituted in 1 ml of 80% ethanol. Extracts were pipetted into microcentrifuge tubes and stored at −80°C until hormone analyses.

### Field-friendly dry extraction

#### Procedures to be conducted in the field

Adapted from Hunt and Wasser (2003), wet faecal samples were thawed, and approximately 1 g (±0.01 g) was weighed and placed into a 15-ml falcon tube with 10 ml of 90% ethanol. Tubes were capped and placed on a rotator (Thermo Scientific, 9420-11-021) for 60 minutes at 30 rpm. Samples were then centrifuged for 30 minutes at 2400*g*. The supernatant was pipetted into a glass tube, placed in a hot water bath (~48°C) and dried using an Evap-o-Rac. Tubes were sealed with parafilm and stored in a dark place at ambient temperature for 7 days.

#### Procedures to be conducted in the laboratory

After 7 days, 1 ml of 80% ethanol was added using a repeater pipette in 0.2-ml increments, then vortexed for 20 seconds. Extracts were pipetted into microcentrifuge tubes and stored at −80°C until hormone analyses.

### Field-friendly wet extraction

#### Procedures to be conducted in the field

Samples were treated the same as the field-friendly dry (FFD) method except that samples were not dried down prior to simulating shipping conditions. Instead, ethanol extracts were left at ambient temperature for 7 days.

#### Procedures to be conducted in the laboratory

After 7 days, ethanol extracts were dried down using an Evap-o-Rac, reconstituted in 1 ml of 80% ethanol, vortexed for 20 seconds and stored at −80°C until hormone analyses.

### Hormone analyses

**Figure 2:**
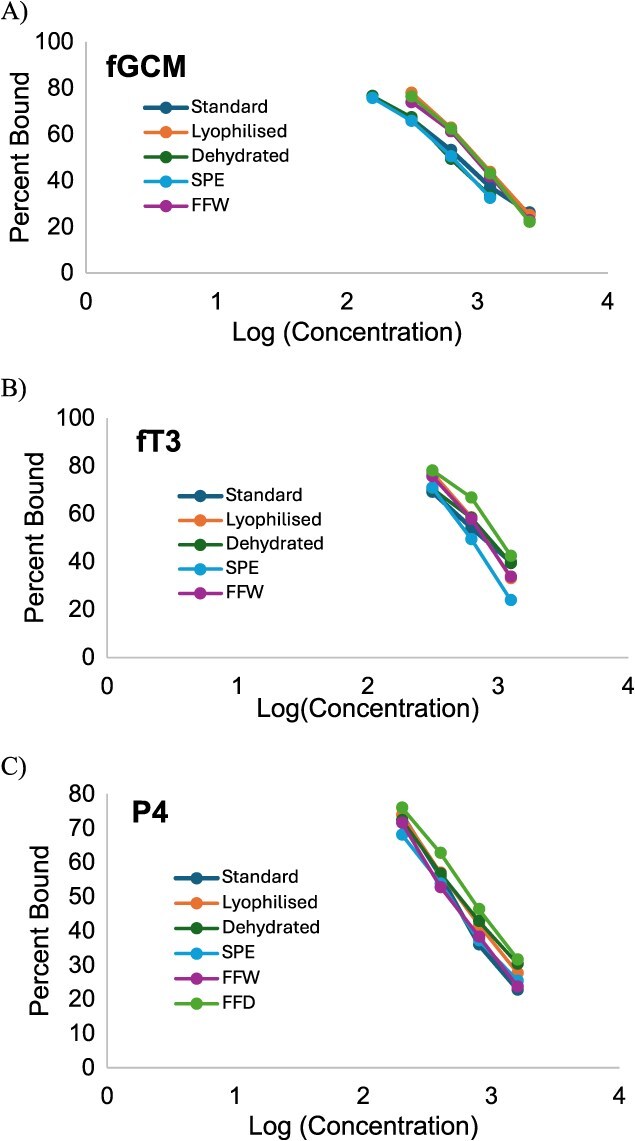
Assay validation results. (A) fGCM concentrations for standards and samples by method. Dilution factors were from 1:2, 1:4, 1:8, and 1:16. (B) fT3 concentrations for standards and samples by method. Dilution factors were from 1:8, 1:16, and 1:32. (C) fPM concentrations for standards and samples by method. Dilution factors were from 1:8, 1:16, 1:32, and 1:64.

Concentrations of fGCM and fT3 were measured by EIAs (Arbor Assays, Ann Arbor, MI, Catalogue # K014-H, K056-H) used previously with elephant faecal extracts (Oduor *et al.,* 2020; Szott *et al.,* 2020), respectively. Extracts for fGCM and fT3 were diluted 1:4 and 1:16 in Arbor Assays buffer, respectively. The EIA for fPM (Arbor Assays, K025-H5) was validated for elephant faecal samples by demonstrating a high correlation (*r* = 0.914) with results from a previously validated EIA that used a monoclonal progesterone antibody (CL425, Coralie Munro, UC Davis, CA) (Freeman *et al.,* 2010). Extracts for fPM were diluted in Arbor Assays buffer at 1:25. Each EIA was validated by demonstrating (i) parallelism between serial dilutions of pooled elephant faecal extracts and the respective standard curve (Fig. 2, Fig. S1) and (ii) significant recovery of standard hormone added to a low pool of faecal extract (Table 1). Only samples that bound between 20% and 80% were used for linear regression analyses. Regardless of the preservation method, all EIAs demonstrated recovery slopes within the acceptable range (0.7–1.2) and *R*^2^ > 0.95 (Keel and Grotjanand, 1996; Ezan and Grassi, 2000), except for the fT3 EIA with the DEHY sample pool, which had a slope of 1.318 (Fig. 2A–C and Fig. S1). A subsample of each faecal sample was dried to permit hormone concentrations to be converted to and expressed as ‘ng/g’ dry weight (DW). Assay sensitivities were 0.02, 0.04 and 0.05 ng/ml for the corticosterone, T_3_ and progesterone EIAs, respectively. All intra- and interassay coefficients were <10% and <15%, respectively.

**Table 1:** Results of accuracy tests for fGCM, fT3 and fPM EIAs by preservation method

Preservation method	fGCM (1:4)	fT3 (1:16)	fPM (1:25)
LYO	Slope = 1.059	Slope = 1.079	Slope = 1.032
	*R* ^2^ = 0.9977	*R* ^2^ = 0.9994	*R* ^2^ = 0.9980
DEHY	Slope = 1.193	Slope = 1.318	Slope = 1.023
	*R* ^2^ = 0.9986	*R* ^2^ = 0.9993	*R* ^2^ = 0.9985
SPE	Slope = 1.144	Slope = 1.048	Slope = 1.073
	*R* ^2^ = 0.9973	*R* ^2^ = 0.9994	*R* ^2^ = 0.9999
FFW	Slope = 1.099	Slope = 1.141	Slope = 1.019
	*R* ^2^ = 0.9981	*R* ^2^ = 0.9977	*R* ^2^ = 0.9990
FFD	SlSlope = 1.083	Slope = 1.197	Slope = 1.127
	*R* ^2^ = 0.9957	*R* ^2^ = 0.9997	*R* ^2^ = 0.9936

### Calculation of hormone concentrations

LYO and DEHY sample concentrations were indexed to DW by the equation as follows:


\begin{align*}=\frac{\mathrm{Concentration}\ \left(\mathrm{pg}/\mathrm{g}\right)\times \mathrm{Dilution}\ \mathrm{Factor}\times \mathrm{Extraction}\ \mathrm{Volume}\ \left(\mathrm{ml}\right)}{\mathrm{Dry}\ \mathrm{extraction}\ \mathrm{weight}\ \left(\mathrm{g}\right)} \end{align*}

For those methods that relied on wet faecal extractions (i.e. field-friendly wet [FFW] and FFD), samples were corrected by the equation as follows:


\begin{align*} =\frac{\mathrm{Concentration}\ \left(\mathrm{pg}/\mathrm{g}\right)\times \mathrm{Dilution}\ \mathrm{Factor}\times \mathrm{Extraction}\ \mathrm{Volume}\ \left(\mathrm{ml}\right)\Big]}{\mathrm{Wet}\ \mathrm{extraction}\ \mathrm{weight}\ \left(\mathrm{g}\right)}\times \frac{\mathrm{Wet}\ \mathrm{faeces}\ \left(\mathrm{g}\right)}{\mathrm{Dry}\ \mathrm{faeces}\ \left(\mathrm{g}\right)} \end{align*}

Final concentrations from SPE cartridges were calculated based on the following equation:


\begin{align*} =\frac{\mathrm{Concentration}\ \left(\mathrm{pg}/\mathrm{g}\right)\times \mathrm{Dilution}\ \mathrm{Factor}\times \mathrm{Cartridge}\ \mathrm{Extraction}\ \mathrm{Volume}\ \left(\mathrm{ml}\right)\times \mathrm{Wet}\ \mathrm{faeces}\ \left(\mathrm{g}\right)}{\mathrm{Wet}\ \mathrm{extraction}\ \mathrm{weight}\ \left(\mathrm{g}\right)\times \mathrm{Cartridge}\ \mathrm{load}\ \mathrm{volume}\ \left(\mathrm{ml}\right)\times \mathrm{Dry}\ \mathrm{faeces}\ \left(\mathrm{g}\right)} \end{align*}

Where:

The concentration (pg/g) is the value provided by the plate reader; the dilution factor is the dilution for each hormone (e.g. 16 if the extracts were diluted at 1:16); the dry extraction weight (g) is the dry faecal powder used for extraction; the extraction volume (ml) is the volume of the aqueous solvent used to extract the faecal solid; the cartridge extraction volume (ml) is the volume used to pull the sample off the cartridge; the cartridge load volume (ml) is the volume of extract loaded onto the cartridge; the wet extraction weight is the weight of wet faeces used for extraction; wet faeces (g) is the weight of wet faeces dried in the field; dry faeces (g) is the final weight of faeces after drying in the field; and the sample volume (ml) is the volume transferred to each well during assay.

### Data analysis

Statistical analyses were performed using SAS (version 9.4). Hormone concentrations from LYO analyses were, a priori*,* considered the ‘true’ faecal concentrations (i.e. gold standard) because LYO removes water to effectively index concentrations (Wasser *et al.,* 1988; Terio *et al.,* 2002). The goal was to ascertain whether the other four methods were reliable measures compared to LYO. Linear mixed models (LMMs) were conducted, with the hormone of interest as the dependent variable and the method the independent variable. The LMMs with repeated measures accounted for the correlation of measures within elephant.  Each preservation technique was compared to LYO using Dunnett’s method to adjust for multiple comparisons. Significance was determined if *P* < 0.05 (two-tailed).

### Ethical considerations

All applicable national and institutional guidelines for the care and use of animals were followed during faecal sample collection. The study was conducted with the approval of the Indianapolis Zoo and Indiana University’s IACUC Committee (19-027).

## Results 

This study analysed the effectiveness of different methods for preserving and storing African savanna elephant faecal samples for fGCM, fT3 and fPM analyses compared to the gold standard LYO technique. Hormone concentrations are presented as mean ± standard deviation on a per gram DW basis (Table 2).

**Table 2:** Mean (±standard deviation) hormone concentrations by preservation method indexed by DW for single faecal samples collected from one male and four female African elephants

Hormone	LYO	DEHY	SPE	FFD	FFW
fGCM (ng/g DW)	24.60 ± 2.24	15.18 ± 2.00*	15.14 ± 3.57*	20.78 ± 2.16	20.36 ± 3.81
fT3 (ng/g DW)	49.13 ± 7.40	73.08 ± 20.8*	23.10 ± 6.71*	40.35 ± 9.26	46.74 ± 18.82
fPM (ng/g DW)	99.12 ± 71.56	92.69 ± 74.09	89.38 ± 64.56	61.85 ± 39.30	71.02 ± 50.37

a Indicates that the technique resulted in concentrations significantly different compared to LYO, *P* < 0.05, for the respective hormone.

### Hormone concentrations by preservation method

**Figure 3:**
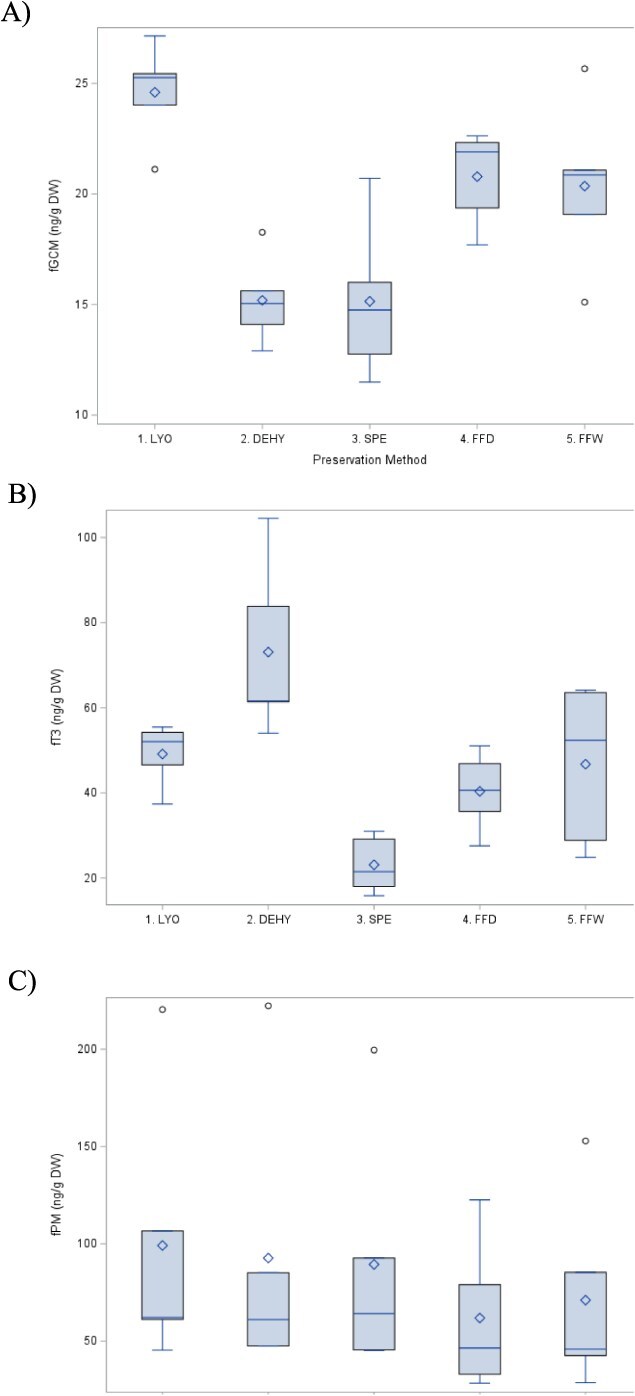
(A) fGCM, (B) fT3 and (C) fPM concentrations by preservation methods. The bottom and top edges of each box represent the range of values between the first and third quartiles. The marker inside the box represents the mean, while the line inside the box represents the median value. The whiskers indicate the range of values outside of the quartile range. Data points whose distances from the interquartile range are greater than 1.5 times the size of the interquartile range are outliers indicated by the open circles. *Depicts a significant difference between the preservation method compared to LYO only (*P* < 0.05).

For fGCM, concentrations were significantly lower for DEHY and SPE methods compared to LYO by approximately 38.3% and 38.4% (*P* < 0.001; Fig. 3), respectively, while faecal samples preserved in FFW and FFD conditions showed no discernible differences compared to LYO (*P* = 0.100 and *P* = 0.152, respectively). For fT3, concentrations were lower for SPE by approximately 53% (*P* = 0.031), while concentrations for DEHY were higher by approximately 48.7% (*P* = 0.050) compared to LYO. FFW and FFD showed no significant differences from LYO for fT3 concentrations (*P* = 0.996 and *P* = 0.719, respectively; Fig. 3). There were no discernible differences by method for fPM concentrations (*P* > 0.742; Fig. 3). The observed differences were apparent despite hormone concentrations being indexed by DW, suggesting that none of the methodologies perfectly mimicked LYO. This may be attributed to differences in alterations rates among methods (Ziegler and Wittwer, 2005; Pappano *et al.,* 2010; Mesa *et al.,* 2014; Crossey *et al.,* 2018; Webster *et al.,* 2018).

### Advantages and limitations by preservation method

Each preservation method has its advantages and limitations. Dehydrating samples requires only a weigh scale that goes to 0.001 g and a food dehydrator, and thus, it is relatively inexpensive to implement and can be done with limited resources. It requires little laboratory experience and training, which can be advantageous when working in under-resourced environments. Further, once the samples are placed in the dehydrator, no further staff time is needed until removal and shipping. Lastly, dry samples are lighter and less expensive to ship. As to disadvantages, smaller solar systems may increase drying time and risk hormone metabolite alteration. The dehydrator needs to be thoroughly cleaned with organic solvents between uses to avoid contamination. Further, samples dried by dehydration (DEHY) demonstrated a slight increase in weight during the 7-day waiting period. The weight increase suggests that water from the surrounding environment was reintroduced to the sample, and such moisture uptake may be exacerbated with poor storage or transport practices. Lastly, although DEHY with a food dehydrator is a viable option, as demonstrated in other species (Postiglione *et al.,* 2022), it resulted in an underestimation of fGCM, similar to what has been observed by others both in savanna elephants and other hindgut fermenters (Osburn *et al.,* 2024), and overestimation of fT3 concentrations.

SPE cartridges, overall, performed the poorest and had the most drawbacks. The main advantages are that they result in ‘cleaner’ samples (i.e. samples contain less debris) that can be stored at ambient temperatures (Palme *et al.,* 2013). However, this method often results in an underestimation of hormone concentrations, especially for fGCM and fT3, perhaps because of inefficient elution of hormones from the column. It also requires extensive laboratory training and equipment, is time and labour intensive (taking upwards of several hours per sample) and relies on expensive consumables.

The FFW and FFD methods results were the most similar to LYO samples for fGCM and fT3, and had no significant difference in fPM concentrations compared to LYO. FFW requires minimal lab supplies and equipment, and relies on extraction with ethanol, which is easier to source in range countries. However, ethanol is classified as a hazardous material, limiting shippable volumes, and so is a major concern for the FFW method when samples must be shipped to outside laboratories. Although FFD requires an apparatus to dry down the samples, it is possible to bring such an apparatus to the field, and it avoids the need to ship ethanol, making it more attractive than FFW.

Based on these findings and field experience, we suggest that FFD would be a desirable preservation alternative given the results and strong benefits associated with this method when shipping is required. Ultimately, field conditions and laboratory infrastructure should be a primary consideration as each method has advantages and limitations.

### Evaluating hormone preservation techniques

This study highlights the importance of measuring multiple hormones from the same sample to comprehensively assess an individual’s physiological state. Previous studies have mostly focused on single hormones, particularly fGCM. For instance, Hunt and Wasser (2003) compared four preservation methods for African elephant fGCM, finding that room temperature storage of lyophilized samples and wet faeces frozen at −20°C led to the most stable fCGM concentrations over 2 years. Lacomme *et al.* (2023) used a food dehydrator and desiccant to process samples similar to the current study and found a 6% reduction in fGCM compared to control samples frozen at −20°C for 12–24 hours and then lyophilized. This was a much smaller reduction in concentration than what was observed in the current study and may reflect differences between dehydrators used. It is important to recognize that results can also vary based on the time elapsed between defecation and sample preservation (Crossey *et al.,* 2018; Webster *et al.,* 2018) and on species and assay used (Lexen *et al.,* 2008).

In conclusion, this study highlights the importance of methodological consistency and transparency in faecal hormone analyses rather than advocating for a single ‘gold standard’ technique. While extraction methods may vary, thoroughly reporting protocols allows the replication and validation of a study. Further, it is important to keep methods the same during a study to retain consistency. Because different methods can lead to different results, it is unwise to compare concentrations across studies, even if methods appear similar, without proper validation. Ultimately, establishing reliable, cost-effective and field-friendly techniques for assessing African savanna elephant physiology is crucial, especially given increasing environmental pressures and the growing need for accurate welfare assessments.

## Supplementary Material

Web_Material_coaf026

## Data Availability

The data underlying this article will be shared on reasonable request to the corresponding author.

## References

[ref1] Chase MJ, Schlossberg S, Griffin CR, Bouché PJC, Djene SW, Elkan PW, Ferreira S, Grossman F, Kohi EM, Landen K et al. (2016) Continent-wide survey reveals massive decline in African savannah elephants. PeerJ 4: e2354. 10.7717/peerj.2354. 27635327 PMC5012305

[ref2] Crossey B, Ganswindt A, Chimimba C (2018) Faecal glucocorticoid metabolite concentrations and their alteration post-defaecation in African wild dogs *Lycaon pictus* from South Africa. Wildl Biol 2018: 1–6. 10.2981/wlb.00469.

[ref3] Edwards KL, McArthur HM, Liddicoat T, Walker SL (2014) A practical field extraction method for non-invasive monitoring of hormone activity in the black rhinoceros. Conserv Physiol 2: cot037. 10.1093/conphys/cot037. 27293621 PMC4732489

[ref4] Ezan E, Grassi J. (2000) Optimization. In Gosling JP, ed. Immunoassays: A Practical Approach. Oxford University Press, Oxford, UK, pp. 95–104, 10.1093/oso/9780199637119.003.0007.

[ref5] Foley CAH, Papageorge S, Wasser SK (2001) Noninvasive stress and reproductive measures of social and ecological pressures in free-ranging African elephants. Conserv Biol 15: 1134–1142. 10.1046/j.1523-1739.2001.0150041134.x.

[ref6] Freeman EW, Abbondanza NF, Meyer JM, Schulte BA, Brown JL (2010) A simplified method for monitoring progestagens in African elephants under field conditions. Methods Ecol Evol 1: 86–91. 10.1111/j.2041-210X.2009.00004.x.

[ref7] Gobush KS, Mutayoba BM, Wasser SK (2008) Long-term impacts of poaching on relatedness, stress physiology, and reproductive output of adult female African elephants. Conserv Biol 22: 1590–1599. 10.1111/j.1523-1739.2008.01035.x. 18759771

[ref8] Hunt KE, Wasser SK (2003) Effect of long-term preservation methods on fecal glucocorticoid concentrations of grizzly bear and African elephant. PBZ 76: 918–928. 10.1086/380209. 14988807

[ref9] Keel HE, Grotjanand BA (1996) Data interpretation and quality control. In EP Diamandis, TK Christopoulos, eds, Immunoassay. Academic Press, San Diego, California, pp 51–95.

[ref10] Lacomme L, Guerbois C, Fritz H, Ganswindt A, Rey B (2023) Validation of a field-friendly faeces drying and storage method for quantifying faecal glucocorticoid metabolites in African elephants (*Loxodonta africana*) opens up new perspectives for conservationists. Conserv Physiol 11: coad053. 10.1093/conphys/coad053. PMC10395557 37538993

[ref11] Lexen E, El-Bahr SM, Sommerfeld-Stur I, Palme R, Mostl E (2008) Monitoring the adrenocortical response to disturbances in sheep by measuring glucocorticoid metabolites in the faeces. Wien Tierarztl Monatsschr 95: 64.

[ref12] Mesa B, Brown JL, Pelican K, Kelly MJ (2014) Effect of natural environmental conditions in Belize on fecal glucocorticoid metabolite concentrations in jaguars (*Panthera onca*). Conserv Physiol 2: 1–10. 10.1093/conphys/cou039. PMC4732494 27293660

[ref13] Oduor S, Brown J, Macharia GM, Boisseau N, Murray S, Obade P (2020) Differing physiological and behavioral responses to anthropogenic factors between resident and non-resident African elephants at Mpala ranch, Laikipia County, Kenya. PeerJ 8: e10010. 33062433 10.7717/peerj.10010 PMC7528812

[ref14] Osburn KR, Crossey B, Majelantle TL, Ganswindt A (2024) A field-friendly alternative to freeze-drying faeces for glucocorticoid metabolite quantification in animals of different feeding classes. MethodsX 13: 103077. 10.1016/j.mex.2024.103077. 39717121 PMC11665413

[ref15] Palme R (2005) Measuring fecal steroids: guidelines for practical application. Ann N Y Acad Sci 1046: 75–80. 10.1196/annals.1343.007. 16055844

[ref16] Palme R, Touma C, Arias N, Dominchin MF, Lepschy M (2013) Steroid extraction: get the best out of faecal samples. Wien Tierärztl Mschrift 100: 238–246.

[ref17] Pappano DJ, Roberts EK, Beehner JC (2010) Testing extraction and storage parameters for a fecal hormone method. Am J Primatol 72: 934–941. 10.1002/ajp.20859. 20623500

[ref18] Pokharel SS, Brown JL (2023) Physiological plasticity in elephants: highly dynamic glucocorticoids in African and Asian elephants. Conserv Physiol 11: coad088. 10.1093/conphys/coad088. 39583302 PMC10673820

[ref19] Postiglione G, Accorsi PA, Ganswindt A, Crossey B (2022) A field-friendly alternative to freeze-drying faeces for glucocorticoid metabolite analyses of African wild dogs (*Lycaon pictus*). MethodsX 9: 101623. 10.1016/j.mex.2022.101623. 35111576 PMC8790624

[ref20] Szott ID, Pretorius Y, Ganswindt A, Koyama NF (2020) Normalized difference vegetation index, temperature and age affect faecal thyroid hormone concentrations in free-ranging African elephants. Conserv Physiol 8: coaa010. 10.1093/conphys/coaa010. 32577285 PMC7297438

[ref21] Terio KA, Brown JL, Moreland R, Munson L (2002) Comparison of different drying and storage methods on quantifiable concentrations of fecal steroids in the cheetah. Zoo Biol 21: 215–222. 10.1002/zoo.10036.

[ref22] Viljoen JJ, Ganswindt A, Palme R, Reynecke HC, Du Toit JT, Langbauer WR Jr (2008) Measurement of concentrations of faecal glucocorticoid metabolites in free-ranging African elephants within the Kruger National Park. Koedoe 50: 18–21. 10.4102/koedoe.v50i1.129.

[ref23] Wasser SK, Papageorge S, Foley C, Brown JL (1996) Excretory fate of estradiol and progesterone in the African elephant (*Loxodonta africana*) and patterns of fecal steroid concentrations throughout the estrous cycle. Gen Comp Endocrinol 102: 255–262. 10.1006/gcen.1996.0067. 8998970

[ref24] Wasser SK, Risler L, Steiner RA (1988) Excreted steroids in primate feces over the menstrual cycle and pregnancy. Biol Reprod 39: 862–872. 10.1095/biolreprod39.4.862. 3207809

[ref25] Webster AB, Burroughs REJ, Laver R, Ganswindt A (2018) Non-invasive assessment of adrenocortical activity as a measure of stress in leopards *Panthera pardus*. Afr Zool 53: 53–60. 10.1080/15627020.2018.1467280.

[ref26] Ziegler TE, Wittwer DJ (2005) Fecal steroid research in the field and laboratory: improved methods for storage, transport, processing, and analysis. Am J Primatol 67: 159–174. 10.1002/ajp.20175. 16163716

