# Supplementary material for: Comparison of methods for faecal hormone preservation and analysis in African savanna elephants under field conditions
Source: Conserv Physiol. 2025 Apr 23;13(1):coaf026. doi: 10.1093/conphys/coaf026 (PMC12060003; doi:10.1093/conphys/coaf026)
Supplement: Web_Material_coaf026 [file web_material_coaf026.zip › field friendly_supplemental file.pdf]

Supplemental File

Comparison of methods for faecal hormone preservation and analysis in African savanna elephants under field conditions

Daniella E. Chusyd*, Department of Environmental and Occupational Health, Indiana University, Bloomington, IN, 47405, USA; dchusyd@iu.edu
Emily Chester, College of Veterinarian Medicine, Auburn University, Auburn, AL, 36849
Tessa Steiniche, Department of Environmental and Occupational Health, Indiana University, Bloomington, IN, 47405, USA
Stephanie Dickinson, Department of Epidemiology and Biostatistics, Indiana University, Bloomington, IN, 47405, USA
Bailey Ortyl, Department of Epidemiology and Biostatistics, Indiana University, Bloomington, IN, 47405, USA
Steve Paris, Smithsonian Conservation Biology Institute, Front Royal, VA, 22630, USA
Nicole Boisseau, Smithsonian Conservation Biology Institute, Front Royal, VA, 22630, USA
Michael Wasserman, Department of Anthropology, Indiana University, Bloomington, IN, 47405, USA
Janine L. Brown, Smithsonian National Zoo & Conservation Biology Institute, Front Royal, VA, 22630, USA

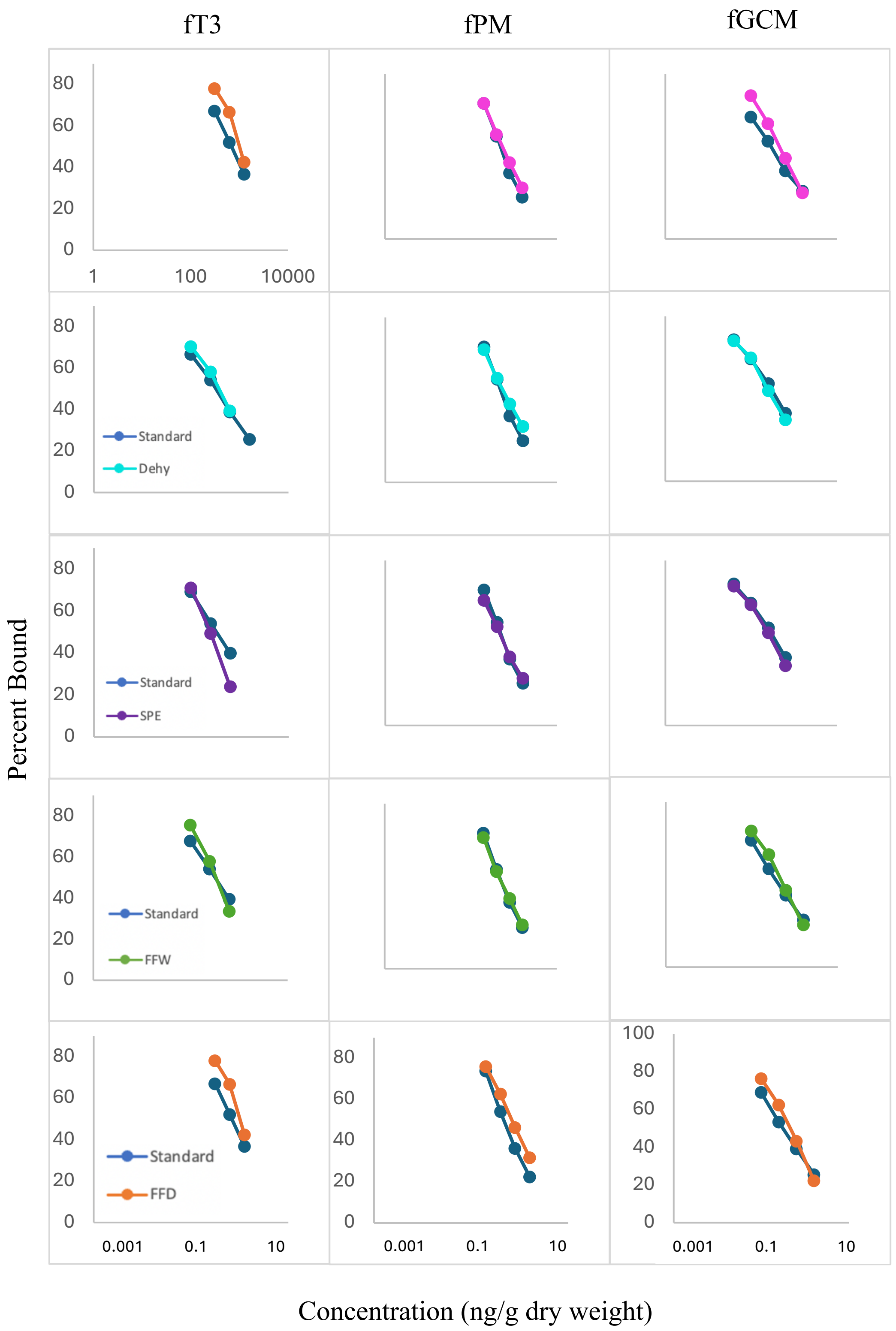

Figure S1. Parallelism for fecal thyroid hormone (fT3; left column), progestogen (fPM; middle column), and glucocorticoid metabolites (fGCM; right column), for each of the five preservation methods.
